# “Socialization for Scarcity” in Emergency Management: Rethinking Assumptions of Resource Scarcity in Humanitarian Crises

**DOI:** 10.5334/aogh.3960

**Published:** 2023-03-24

**Authors:** Zoe Fanning

**Affiliations:** 1 London School of Economics and Political Science, London, UK; 2 Mayo Clinic Alix School of Medicine, Rochester, MN, US

**Keywords:** humanitarian crises, socialization for scarcity, resource scarcity, emergency management, global health

## Abstract

**Background:**

Physician-anthropologist Paul Farmer theorizes a process of “socialization for scarcity” (SfS), which assumes permanent and unchangeable resource scarcity for the world’s poor. International health and poverty decisions that are based off of this premise are therefore used to justify inadequate care for vulnerable populations.

**Objectives:**

The theory of SfS has predominantly been applied to the context of global health and development. This paper aims to apply SfS to the field of emergency management, asking, “How does SfS function in the context of humanitarian crises, and what implications does this have for emergency management?”

**Methods:**

This paper reviewed Farmer’s own descriptions of SfS as well as articles by colleagues and other scholars who elaborated on his theory, analyzing their contributions to issues relevant in emergency management.

**Findings:**

This review finds that SfS is both applicable to and amplified within emergency management because of the uncertain, competitive, and urgent nature of humanitarian crises. The paper then describes potential approaches to combating SfS in emergency contexts.

**Conclusions:**

SfS is the result of deficient effort toward discovering approaches to managing emergencies that do not presume scarcity. The assumption of permanent resource scarcity, especially in low- and middle-income countries (LMICs), is a matter of inequity and injustice and stands opposed to imperative systemic change. Emergency managers must work to eradicate dangerous presumptions that leave already suffering individuals even further from the dignified, appropriate and adequate care they require and deserve.

*Scarcity for ourselves? No. Scarcity for our mom? No. For our own kids? No. We’re socialized for scarcity for other people, and they’re usually black or brown or poor. So then we start cutting corners*.

**Paul Farmer, Physician-Anthropologist** [1]

## Introduction

When crises emerge, vulnerable individuals bear the disasters’ greatest brunt: those who live in poor housing, work in subsistence farming or the informal sector, or experience poverty and chronic illness and those whose families and communities have endured these plights and more for decades and centuries. Vulnerable communities, especially those in low- and middle-income countries (LMICs), also tend to have fewer resources to prepare for, mitigate, and rebuild from emergencies. The extent to which this dearth of resources is perceived as permanent, however, has significant implications for emergency management.

Coming primarily from the field of global health, physician-anthropologist Paul Farmer theorizes a process of “socialization for scarcity” (SfS) that is used to justify inadequate care for vulnerable populations. This paper applies this theory to emergency management and finds that SfS is both applicable to and amplified within humanitarian crises for three primary reasons: (1) the uncertain nature of emergencies; (2) competition for “market” share and profit motives within the humanitarian system; and (3) the urgent need for humanitarian assistance. I do not argue against the undeniable fact that many countries and communities have fewer resources than others but rather the extent to which this is taken to be certain, fixed, and unavoidable.

This paper begins with a brief literature review before further defining Farmer’s SfS. Using a reflective approach, I then analyze this theory within the context of emergency management and discuss assumed permanence of scarcity as the product of SfS. Finally, I end with suggestions for ways forward.

## Literature Review

Common within emergency management literature is discussion of resource availability and allocation, which it often describes as a primary, reoccurring challenge [2,3,4,5,6]. Further, literature specific to “resource-poor” settings and LMICs tends to imply homogeneity and frequently takes resource scarcity as its assumed starting point for emergency management [2,7]. Some authors question inequitable resource availability, including Schrecker [8,9], who asks why some places have fewer resources than others and how this uneven distribution may be “denaturalized,” and Scoones et al. [10], who posit the existence of “political scarcity” in addition to absolute and relative scarcity. These authors give valuable insights into the historical and contemporary routes through which some communities experience enduring resource poverty. Few authors, however, dedicate time to asking why and how this scarcity is often assumed to begin with [11,12].

Emergency management literature that is reflective in nature occasionally highlights the ability of social construction to build narratives of and responses to crises and even to determine the paths of the crises themselves. These include Weick’s “enacted sensemaking” and Nowell and Steelman’s exploration of embeddedness in disaster responses [13,14]. This literature highlights an important component of emergency management and relief, namely its interaction with social structures, institutions, biases, and cognition.

Available literature on SfS, discussed further in the following section, is predominantly focused on case studies of global health and development efforts [15,16,17]. As far as this author is aware, application of the specific theory of SfS to emergency management is sparse. There are many similarities between the global health field and the humanitarian field that justify analysis of SfS in emergency management, including Western influence and power, the drive to alleviate suffering, and international interaction. With knowledge of this gap and the need for greater discussions of assumed scarcity and its impact on vulnerable populations in emergency management literature, this paper asks, “How does SfS function in the context of humanitarian crises, and what implications does this have for emergency management?”

## Paul Farmer’s “Socialization for Scarcity”

Farmer’s SfS is defined as “the assumption that resources for poverty reduction and international health initiatives will be in perpetually short supply” [18(p61)]. Broadening how we make sense of “resource scarcity” within humanitarian crises is therefore consequential [13]. The “resources” referred to in most literature on SfS are primarily material in nature [17], but this understanding can be expanded to include Farmer’s “5 S’s”: staff, stuff, space, systems, and social support [19,20]. This paper utilizes the latter description because it more comprehensively captures the impact of SfS on emergency management.

As its name suggests, socialization and normalization of resource scarcity is derived from social interaction rather than constituting an innate characteristic of the global health system: “‘Socialization’ implies that notions of scarcity are acquired, un-tame, unfixed—and therefore modifiable. Socialization is an event … that allows for the maintenance of a state of affairs through the twin vehicles of narrative and expectation” [17(p22)]. SfS creates a perspective through which the starting point for global health efforts and development projects in LMICs is scarcity of resources. This is taken to be an unchangeable and fundamental assumption. The term “LMICs” itself has, after all, come to be understood as synonymous with “resource poor.” This presumption then frames the problem and narrows available solutions, often leading to substandard results.

Further, SfS can set up false dichotomies, “pitting one good thing against another—prevention versus treatment, nurses versus doctors, emergency response versus development. It’s a curious pathology that comes from us” [21]. This unnecessary opposition is further explained by Greene et al., who note that, because of this constructed pathology, “administrators of development projects were frequently forced to choose between immediate provision of services and long-term investment in infrastructure” [18(p61)]. As a result, lives of already vulnerable individuals are put even further at risk.

## Amplification of SfS in Times of Crisis

SfS can provide significant insight into emergency management. Humanitarian emergencies are often described as “wicked problems” due to their heightened uncertainty, intractability, and complexity and their diversity of stakeholders [22,23]. Further, wicked problems can be perceived and defined in numerous, consequential ways, and this “choice of explanation determines the nature of the problem’s resolution” [24(p166)]. In the context of emergencies, where accurately understanding the crisis at hand is imperative to implementing effective relief efforts, this implies that emergency managers’ perspectives on crises can have substantial consequences for those in need of assistance.

Emergencies bring obvious logistical challenges that are usually accompanied by threats or realizations of resource scarcity, but it is vital to separate the *actual* logistical and scarcity issues from those that are *constructed*. In essence, resource scarcity—and potentially the development of major crises themselves—may be brought about by assumptions of permanent resource scarcity [13]. These expectations of unmodifiable scarcity can therefore produce self-fulfilling prophecies, making the situation more difficult to address than it may have otherwise been. For Darcy et al. [25], one’s conception of available decision-making frameworks, the values and assumptions one applies, and the mental models one uses to process external information are equally as influential in humanitarian decision-making as that information itself.

With this foundational assumption of unchangeable resource scarcity, it is practically impossible to imagine and pursue a crisis response where the care of vulnerable individuals, especially those in LMICs, is not in some sense inadequate. Assumptions and commitments to them can produce blind spots that prohibit emergency managers from conceptualizing and implementing a potentially more appropriate (i.e., adequately meeting true needs) response [13]. When paired with the danger of scarcity’s assumed permanence, three main overlapping characteristics of emergencies highlight their wicked nature and heighten the phenomenon of SfS. These are explored in the proceeding subsections.

### 1. Uncertainty

Humanitarian emergencies are highly uncertain events. As a result, emergency managers who have been socialized for scarcity may wrongly assume that the proper relief approach is a large influx of goods, when, instead, “the key idea is that you need the right and the precise quantity of selected goods in the right timing and in the right place” [26]. In the context of the ongoing humanitarian crisis in Ukraine, individuals and organizations around the world have sought to aid suffering Ukrainians by sending medical supplies, clothing, and nonperishable food items, among other material goods [27,28]. VanRooyen, the director of the Harvard Humanitarian Initiative, notes, “Having been on the receiving side [of material donations] dozens of times, … they rarely are the right kind at the right place at the right time for the right population” [28]. The substantial needs of those impacted by crises need to be met not with an indiscriminate barrage of items but with pertinent, appropriate aid. SfS stands in the way of this relevancy, highlighting instead the need for simply *more*.

Furthermore, the inherent uncertainty of emergencies complicates supply chain management (SCM) and can amplify the socialized presumption of permanent scarcity. In times of crisis, characteristics of humanitarian SCM differ dramatically from its business counterpart [5]. Within the former, needs and wants are more dire, infrastructure is often impeded or destroyed, and the supply chain’s goals focus more on minimizing suffering rather than cost. These characteristics can each contribute to a demand-supply mismatch, intensified by SfS, by emphasizing the need for *more* goods to combat suffering and overcome debilitated infrastructure. This can result in a convergence of resources with potentially serious consequences, including the administration of expired medications, depression of local markets, and reliance on external assistance [29]. These consequences beg the question: Is there really unavoidable scarcity; or is aid merely inappropriately targeted?

### 2. Competition

The consequences of SfS are further amplified by the humanitarian “marketplace.” Competition among humanitarian stakeholders heightens power dynamics and disincentivizes partnership [30], thus subordinating the needs of crisis victims. This has important implications for emergency management because partnership and cooperation among stakeholders is vital for nonredundant responses.

The humanitarian marketplace contributes to heightening SfS’s impact in two ways. First, by leading humanitarian organizations to compete against one another for donor funding, marketization of the humanitarian system constructs a zero-sum game in which funding and resources needed for relief are perceived as immutably limited. Second, competition among humanitarian organizations can lead to redundancies in relief efforts and “consumes resources that could be spent on problem solving” [30(p6)]. This resultant decrease in available resources can lead to believing that those limitations are an inherent and unchangeable part of the humanitarian system rather than something cooperation could have avoided or mitigated. Farmer aptly remarks that “there would be less scarcity if we all worked together” and that SfS is largely driven by “competing against each other instead of adding up to more than the sum of our parts” [1,21]. This is evidenced by the significant increase in the number of aid organizations responding to crises around the globe in the past three decades, with some scholars arguing that this has contributed to competition, uncertainty, and insecurity within the field rather than a more global civil society, as some might infer [31(p6)]. The rapid expansion in aid organization density and competition for increasingly earmarked donor funding has therefore incentivized inadequate, unequitable responses for those in need. Growing competition has also driven the pursuit of professionalization and specialization within the humanitarian community, with the adverse consequence of making it more and more difficult to maintain an open and innovative mind to response options [25].

Since markets are “social mechanism[s] in which everything has a price,” a marketized humanitarian system “leads us inexorably towards calculations of utility and exchange” [32(p99)]. These ideas and practices have contributed to worsening SfS in emergency management. Within the context of top-down, donor-driven humanitarianism, SfS prompts the assumption that communities will be eternally “resource poor,” and thus any substantial effort toward addressing the root causes of scarcity is unprofitable and wasteful:

[Cost-effectiveness thinking] maximizes utility after assuming scarce resources. This assumption—the often misleading first principle of cost-effectiveness—can yield determinations that simply reflect and reproduce global inequalities rather than allocating resources based on the hierarchy of needs. The poor get cheap care, if they are lucky enough to have access to care at all. [16(p250)]

When combined with a cost-effectiveness calculus, SfS can greatly hinder justification of adequate assistance for victims of humanitarian crises, especially given their “wicked” nature. Further, the profit motive of philanthro-capitalists [33,34] can amplify the effects of SfS by steering relief agendas away from substantially countering resource scarcity and instead contributing to the perception of scarcity as permanent.

### 3. Urgency

Whether an emergency is slow to onset, like drought, or rapid, like many conflicts, the needs of individuals enduring these hardships require urgent assistance. By decreasing the available time for needs assessments and planning, emergency managers are more likely to rest on their assumptions rather than seeking information to dispel or confirm them:

Decision makers with limited time to make decisions tend to rely heavily on the judgment of people they trust, both in constructing the humanitarian “narrative” for a given crisis and in defining response options. This can result in a relatively unchallenging attitude to proposals and evidence used to support them. [25(p7)]

As a result, urgency can amplify SfS by causing response planners to assume inevitable and unavoidable scarcity. This construction of the emergency’s narrative is entangled with Weick’s concept of capacity in crisis sense making, whereby people see and react to crises “that they feel they have the capacity to do something about” [13]. If humanitarians are socialized for scarcity, they are likely to view this resource poverty as insurmountable and are thus less likely to implement an equitable, appropriate response.

The 2014–2016 Ebola outbreak in West Africa, which represented the overlap between global health crises and humanitarian emergencies as a World Health Organization–designated “public health emergency of international concern” (PHEIC) [46], illustrates the pervasion and impact of SfS in urgent crises. Most prior Ebola outbreaks occurred in rural communities, where “the public-health people didn’t much care if mortality approached a hundred percent, as long as they could make sure everyone was accounted for on a contact list” [35(p63)], but the 2014 outbreak reached major cities and crossed national borders. The virus spread quickly, making contact tracing more challenging and threatening many more lives. Emergency and public health responders made urgent decisions that were heavily informed by the assumption that strict disease control efforts should supersede attention to care and context. These judgments were marred by SfS; Farmer recalls how “roiling debates about the quality of care proceeded as if West African clinical deserts were somehow immutably arid and suddenly and forever cut off from the rest of the globe” [35(p32)]. Combining SfS with the World Health Organization’s rarely dispensed designation of the outbreak as a PHEIC and the urgent need to respond to the virus’s quick dissemination led to the development of a “control versus care” paradigm—“isolation without proper care” [35(p64)]—which contributed instead to the disease’s spread and devastation. The socialized assumption that proper care would be impossible in places as “arid” and “forever cut off” as Guinea, Sierra Leone, and Liberia was largely to blame.

As a consequence of concurrent SfS and urgency, fewer participants are involved in the management process, perspectives are narrowed, responses are less detailed, and individuals in need of assistance are not provided the care they innately deserve. Vesely describes these consequences of urgency when paired with scarcity in emergency management:

Ideally, planners would consider a broad spectrum of scenarios, recommend approaches that would be taken when unlimited time and resources are available… . However, the usual scarcity of time and lack of material resources make it impossible to bring all necessary participants into the more detailed type of planning structure needed to consider and implement the actions required to counter the dangers that must be confronted. [36]

SfS implies that the “lack of material resources” Vesely refers to is assumed to be a permanent issue in any LMIC crisis setting, and thus the “ideal” situation is understood as impossibly attainable, as he suggests. However, it does not have to be.

## Addressing SfS in Emergency Management

The National Planning Branch chief of the US Federal Emergency Management Agency (FEMA) recently wrote, “The century-long trend to simply deliver more resources more effectively and more equitably cannot be sustained indefinitely. … It is simply an admission that, at some point, there will not be any more resources to provide” [37]. While Roller appropriately recognizes that it is not simply *more* resources that will address needs amid emergencies, his language concerningly alludes to sweeping claims of permanent scarcity. Daoud employs a model that counters perspectives on unavoidable scarcity such as Roller’s:

Scarcity or abundance as an event arises when goods (A) are quantitatively related to human needs (R)… . According to the Mengerian model, frustration of needs … occurs in two situations: (a) an event of scarcity (R > A); and (b) an event of quasi-scarcity, that is, people are somehow excluded despite the quantitative relation R < A, abundant goods. [11]

This “quasi-scarcity” is especially problematic. In 1981, Amartya Sen wrote that starvation is “the characteristic of some people not *having* enough food to eat. It is not the characteristic of there *being* not enough food to eat” [38]. Beyond disallowing individuals from realizing that resources in disaster settings may not be as inevitably limited as they presume, SfS also hinders recognition of consequential issues relevant to managing the emergency at hand: colonial legacies, political contexts, power dynamics, and social and cultural factors. Quasi-scarcity can also impact the safety of humanitarian workers by forcing them to make difficult resource allocation decisions aimed at reaching those most in need, but that may be perceived by some as defying the humanitarian principle of neutrality [2].

Available literature reveals a number of broad recommendations for combating SfS. One often-repeated suggestion is partnership across stakeholders in emergencies, especially that which employs pragmatic solidarity and produces value creation and knowledge sharing [[Bibr B5][Bibr B39][Bibr B40]]. Additionally, Farmer argues, “The most compelling thing to fight socialization for scarcity on behalf of others is health system strengthening. Health systems that integrate prevention and quality care” [1]. For the field of emergency management, this translates into humanitarian system strengthening that integrates anticipatory planning and prevention with care that assesses and addresses, rather than assumes, needs. Similarly, emphasizing a human right to humanitarian assistance, as has likewise been done by global health advocates in emphasizing a right to health, can help in opposing the complacency that accompanies SfS [17].

Lastly, certain tools of digital humanitarianism, when appropriately applied, can counter the notion that it is too cost-ineffective to adequately address needs due to perceived resource scarcity in LMICs by providing inexpensive, coordinated, clarifying alternatives to traditional response tools [41,42]. Research that expands on the recognition of SfS in emergency management should seek to understand the impact of SfS on the agency, social capital, and capacity of those impacted by humanitarian crises. Further, additional research should build on this paper’s expansion of SfS into emergency management by applying the theory to particular case examples and seeking greater explication of the benefits of expunging SfS from the field.

## Conclusion

While SfS has been deemed a result of inadequate “moral imagination” [43], the ability to empathize with others, I would argue it is also the result of deficient practical imagination, or the desire to dedicate one’s self toward discovering approaches to managing emergencies that do not presume scarcity. The assumption of permanent resource scarcity, especially in LMICs, is a matter of inequity and injustice and stands opposed to imperative systemic change [17]. The uncertain, competitive, and urgent nature of humanitarian crises amplifies the consequences of SfS, which can have the deleterious impact of justifying inadequate relief efforts; when SfS is at play, “management of time, affection, food, water, and family crises (including illness) all fit into this ancient framework of too much and too little” [15(p312)].

Christian et al. contend that allocation strategies in disaster settings “vary greatly depending on whether resources are plentiful or scarce” [44]. Importantly, allocation strategies also vary greatly depending on whether resources are *assumed* to be *permanently* plentiful or scarce. As Farmer described in the dual humanitarian-health crisis prompted by the 2014–2016 Ebola outbreak in West Africa, “This was clinical nihilism: the tools and funding required to improve supportive and critical care, we heard every damn day, were not in the budget. But if Ebola wasn’t in the budget, didn’t that mean the budget, not the virus, was wrong?” [35(p62)]. If SfS is both recognized and actively resisted within the field of emergency management, crisis responses will be less redundant, competition will subside, and resources will be freed up to both meet needs and work against assumptions of permanent scarcity. As a result, responders will be increasingly incentivized to partake in imaginative, contextually informed, and equitable approaches to crises. Emergency managers must avoid becoming prisoners of “circles of certainty” [45(p39)] by eradicating dangerous presumptions that leave already suffering individuals even further from the dignified, appropriate, and adequate care they require and deserve.

## Data Accessibility Statement

All authors had access to the data and a role in writing this paper.
